# Is pre-operative heart rate variability a prognostic indicator for overall survival and cancer recurrence in patients with primary colorectal cancer?

**DOI:** 10.1371/journal.pone.0237244

**Published:** 2020-08-20

**Authors:** M. T. A. Strous, A. M. Daniels, F. M. Zimmermann, F. N. van Erning, Y. Gidron, F. J. Vogelaar

**Affiliations:** 1 Department of Surgery, VieCuri Medical Centre, Venlo, The Netherlands; 2 Department of Cardiology, Catharina Hospital, Eindhoven, The Netherlands; 3 Department of Research, Netherlands Comprehensive Cancer Organisation, Utrecht, The Netherlands; 4 Faculty of Welfare and Health, University of Haifa, Haifa, Israel; University of Oslo, NORWAY

## Abstract

**Background:**

Heart Rate Variability (HRV) represents efferent vagus nerve activity which is suggested to be inversely related to fundamental mechanisms of tumorigenesis and to be a predictor of prognosis in various types of cancer. HRV is also believed to predict the occurrence and severity of post-operative complications. We aimed to determine the role of pre-operative HRV as a prognostic factor in overall and cancer free survival in patients with colorectal cancer.

**Methods:**

Retrospective analysis was performed in a detailed dataset of patients diagnosed with primary colorectal cancer between January 2010 and December 2016, who underwent curative surgical treatment. HRV was measured as time-domain parameters (SDNN (Standard Deviation of NN-intervals) and RMSSD (Root Mean Square of Successive Differences)) based on pre-operative 10 second ECGs. Groups were created by baseline HRV: Low HRV (SDNN <20ms or RMSSD <19ms) and normal HRV (SDNN ≥20ms or RMSSD ≥19ms). Primary endpoints were overall and cancer free survival.

**Results:**

A total of 428 patients were included in this study. HRV was not significantly associated with overall survival (SDNN <20ms vs SDNN ≥20ms:24.4% vs 22.8%, adjusted HR = 0.952 (0.607–1.493), p = 0.829; RMSSD <19ms vs RMSSD ≥19ms:27.0% vs 19.5%, adjusted HR = 1.321 (0.802–2.178), p = 0.274) or cancer recurrence (SDNN <20ms vs ≥20ms:20.1% vs 18.7%, adjusted HR = 0.976 (0.599–1.592), p = 0.924; RMSSD <19ms vs ≥19ms, 21.5% vs 16.9%, adjusted HR = 1.192 (0.706–2.011), p = 0.511). There was no significant association between HRV and CEA-level at one year follow-up, or between HRV and occurrence of a post-operative complication or the severity of post-operative complications.

**Conclusions:**

Heart rate variability was not associated with overall or cancer free survival in patients with primary colorectal cancer who underwent curative surgical treatment. These results do not align with results found in studies including only patients with advanced cancer, which suggests that there is only an association in the other direction, cancer causing low HRV.

## Introduction

In 2018 there were over 1.8 million newly diagnosed colorectal cancer patients worldwide and over 14,000 in the Netherlands alone. It is the fourth most common cause of death worldwide [1,2]. To improve survival it is of importance to get a better insight into modifiable prognostic factors. Emerging evidence suggests that vagal nerve activity, indexed by heart rate variability (HRV) could be one of these prognostic factors [3–7].

HRV is the physiological phenomenon of the fluctuation in time intervals between adjacent heartbeats and represents efferent vagus nerve activity to the heart [7–9]. It has been suggested that efferent vagal activity is inversely related with fundamental mechanisms of tumorigenesis as inflammation, oxidative stress and excessive sympathetic activity [7]. These mechanisms are believed to be controlled by the vagus nerve via a bi-directional brain-to-immune pathways through the release of neurotransmitters via the cholinergic anti-inflammatory pathway [10,11]. A higher vagal tone may reflect a more flexible top-down regulation of the immune-system and physiological activity moderated by the brain [12]. Absence of vagus activity due to vagotomy has been shown to increase the risk of developing colorectal cancer [3].

In addition to influencing development of cancer, vagus nerve activity seems to be a predictor of prognosis in various types of cancer. Recent studies show an association between decreased activity of the vagus nerve and worse survival in patients with cancer of the gastrointestinal tract, liver, pancreas, lung, prostate and breast among others [3,4,7]. Also, patients with normal HRV seem to live longer in different sorts of metastatic cancer, independent of confounders [13]. In patients with colorectal cancer a low HRV at baseline has shown to be associated with higher CEA levels at 12 months after diagnosis, which predicts a poorer prognosis [14].

In patients undergoing curative treatment for colorectal cancer, HRV does not only seem to influence cancer prognosis. A recent study showed that patients with lower HRV have more intraoperative blood loss and more, and more severe, postoperative complications [15].

Identifying patients with low HRV is easy and non-invasive. When its predictive value for the prognosis of cancer patients is of satisfactory significance, vagus nerve activation prior to or during cancer treatment could theoretically be beneficial in improving prognosis [16]. Also, if we could predict the occurrence and severity of postoperative complications based on HRV, improving HRV before surgery could possibly accelerate postoperative recovery and indirectly affect patients’ prognosis. Recent studies focussing on improving HRV by improving physical fitness by means of physical exercise show promising results in both older men and woman [17,18]. However, the only previous study on colon cancer and HRV including patients receiving curative treatment included a small sample and did not examine whether HRV predicts survival in these patients [14]. To clarify the predictive value of HRV in prognosis of patients with colorectal cancer, further exploration is needed. Current studies identifying HRV as a prognostic factor did not specifically focus on colorectal cancer, have small study populations, did not correct for confounders and mainly focused on metastatic disease [3,7–11].

The aim of this study was to determine the role of pre-operative HRV as a prognostic factor in overall and cancer free survival, in patients with primary colorectal cancer who underwent curative surgical treatment.

## Methods

### Data collection

Data from the Netherlands Cancer Registry (NCR) were used. The NCR collects data on all newly diagnosed cancer patients in the Netherlands. Information on patient and tumour characteristics, diagnosis and treatment is routinely collected from the medical records by trained administrators of the cancer registry. Anatomical site of the tumour is registered according to the International Classification of Diseases -Oncology. The tumour-node-metastasis (TNM) classification is used for stage notification of the primary tumour, according to the edition valid at time of cancer diagnosis. Quality of the data is high due to thorough training of the registration team and consistency checks [19].

For the study population, additional data were collected from the medical records of the patients. This encompassed information on HRV, CEA-levels, ASA- classification, comorbidities identified at admission divided into groups (cardiac disease, hypertension, diabetes mellitus, thyroid disease, pulmonary disease, vascular disease, neurological disease and other), occurrence and severity of postoperative complications and cancer recurrence. Groups of comorbidities were chosen based on matching features within these groups and their potential influence on HRV or the endpoint being analysed. Severity of the postoperative complications according to the Clavien-Dindo classification was also documented. Medical records were assessed between January 2019 and July 2019, and re-evaluated for revision of this article between the 20^th^ and 25^th^ of April 2020.

This study was approved by the research committee and the Board of Directors of VieCuri Medical Centre. Data was obtained under the law ‘scientific research and statistics in the interest of public health, where asking for permission is not possible or appropriate for several reasons’ in the Netherlands, unless patients objected to use of their personal medical record for scientific research. Data was encrypted with an encryption key provided by the NCR. Encryption was shortly lifted to access the patients’ number for accessing his/her medical record. Following extraction data were encrypted again.

### Study population

The study population included all consecutive patients diagnosed with primary colorectal adenocarcinoma between January 2010 and January 2017 at VieCuri Medical Centre who underwent curative surgical treatment. Patients with metastatic disease at time of surgery or carcinoma in situ were excluded as their treatment and prognosis differs from those receiving curative treatment for colorectal cancer. Metastasis found within 3 months after surgery were considered present at time of surgery and therefore excluded. Other excluded patients were: patients with neuroendocrine tumours because of different tumour characteristics and prognosis, patients with cardiac arrhythmias (including atrial and ventricular extrasystole), pacemakers, patients taking beta-blockers as this enhances HRV indexes, or patients with bradycardia (heart rate <50 bpm) or tachycardia (heart rate >110 bpm) as this precluded reliable calculation of HRV [20]. We did not exclude patients taking alpha-blockers, calcium-inhibitors, diuretics, amiodarone, ACE-inhibitors or ARB’s as these types of medication reduce central sympathetic functioning rather than peripheral and their influence on HRV is therefore less univocal and sometimes completely absent [21,22].

### Heart rate variability

Heart rate variability was analysed using a 12-lead 10-second ECG (150Hz) used for pre-operative screening. In case of multiple ECG’s per patient the most recent ECG before date of surgery was used for HRV-analysis. In case of multiple ECG’s per patient on the same date the ECG with the best quality was chosen, meaning an ECG without motion artefacts. In case of motion artefacts there was always an ECG without motion artefacts available recorded on the same date. Time between two consecutive R-peaks was measured in lead II with an accuracy of 0.2ms using MUSE-ECG. HRV was presented using the time-domain HRV parameters SDNN (Standard Deviation of NN-intervals) and RMSSD (Root Mean Square of Successive Differences) in milliseconds, calculated using the following calculations [23]:

$$\mathrm{SDNN}=\sqrt{\frac{1}{n-1}\sum_{i=1}^{n}{\left(RR_{i}-RR_{\mathrm{mean}}\right)}^{2}}$$ (1)

$$\mathrm{RMSSD}=\sqrt{\frac{1}{n-1}\sum_{i=1}^{n-1}{\left(RR_{i+1}-RR_{i}\right)}^{2}}$$ (2)

SDNN and RMSSD obtained from 10s ECGs were found to correlate with results of ECGs of longer durations. Power spectral analysis HRV parameters as LF and HF can only be obtained in longer recording ECGs and were therefore not implementable in this study [20,23,24].

SDNN and RMSSD were both analysed as continuous variables as well as binary variables using cut-offs of <20ms versus ≥20ms and <19ms versus ≥19ms respectively. In case of an SDNN <20ms or RMSSD <19ms HRV was classified as low, and in case of SDNN ≥20ms or RMSSD ≥19ms as normal. These cut-off values were based on cut-off values used in other studies showing an association between low-HRV as SDNN <20ms and RMSSD <19ms and (colorectal) cancer, as there is no standardised definition of low and normal HRV [5,7,14].

### Endpoints and definitions

The primary endpoints of this study were overall and cancer free survival. Overall survival was defined as the time between the date of surgery to the date of death or last follow-up date in months. Cancer free survival was defined as the time in months from the date of surgery until the date of cancer recurrence (defined as the first date of either radiologic or pathologic diagnosis of metastases or tumour recurrence of colorectal cancer). Patients dying without cancer recurrence were censored on day of death. Secondary endpoints were elevated CEA-level (>5,0 ug/l) at one-year follow-up, occurrence of post-operative complications within 30 days after surgery and severity of post-operative complications according to the Clavien-Dindo classification.

### Statistical analysis

In this retrospective observational cohort study we utilized descriptive statistics to provide an overview of control variables of the study population (patient characteristics as age, sex, BMI, comorbidities and ASA-classification, heart rate and tumour characteristics as TNM-stage, tumour localisation and tumour differentiation) and their association with HRV and prognosis. Normal distribution of the continuous variables heart rate, age and BMI as well as SDNN and RMSSD were tested with a Kolmogorov-Smirnov test. Because of normal distribution heart rate, age and BMI were compared between HRV-groups using unpaired t-test. All other variables were categorical and were compared between HRV-groups using Chi-square statistics as groups were all of sufficient power.

Differences in overall survival and cancer free survival in months according to SDNN and RMSSD were visualized by means of Kaplan-Meier curves and statistically tested using the log-rank test. Multivariate cox-regression analyses were conducted to calculate the prognostic association between HRV and overall and cancer free survival, while adjusting for other prognostic variables. Multivariate logistic regression was used to assess the independent effect of SDNN and RMSSD on CEA-levels and the occurrence- and severity of post-operative complications. Variables included for adjustment were chosen by means of forward stepwise selection based on clinical judgment, differences at baseline (e.g., differences on any predictor between patients who later died or not) and database availability, and depended on the analysed endpoint. Those included patient demographics (age, sex, body-mass-index, comorbidities identified at admission divided into groups (cardiac disease, hypertension, diabetes mellitus, thyroid disease, pulmonary disease, vascular disease, neurological disease, other (including Crohn’s disease, hepatitis, kidney failure disorders, anaemia, depression, arthritis)), tumour characteristics (localisation, stage, differentiation) and the occurrence of post-operative complications, when the later was not an outcome. Differences in CEA-level at baseline and one year check-up between and within groups of low HRV and normal HRV were assessed with a repeated measures linear model and tested using the tukey test. To test the implication of a longer time between ECG and treatment all analyses were repeated after excluding patients with an ECG older than 6 months. A two-tailed p-value ≤ 0.05 was considered significant in all analyses. Data were analysed using IBM SPSS Statistics, version 25.0 (IBM Corp, NY, Armonk, USA).

## Results

**Fig 1:**
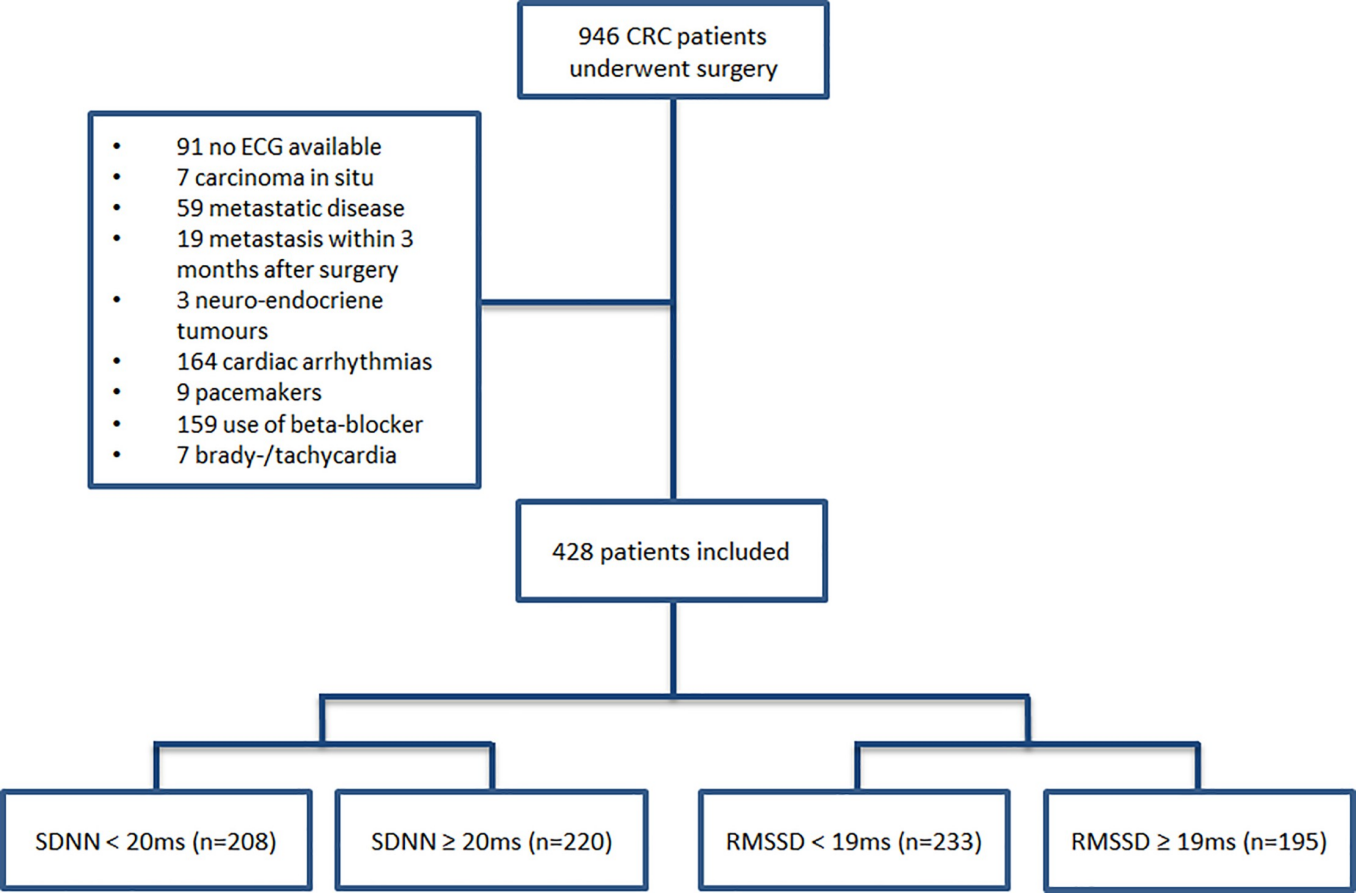
Flowchart of the study.

Of 946 colorectal cancer patients that underwent a surgical resection, a total of 428 patients were included in this study. Reasons for exclusion are presented in Fig 1. Median SDNN and RMSSD were 20.4ms (interquartile range (= IQR) 11.5ms-35.1ms) and 17.5ms (IQR 9.9ms-29.9ms), respectively. Table 1 shows descriptive data of the included patients by HRV groups. Baseline heart rate and age were negatively associated with HRV. The group of patients with low HRV contains more patients with a history of cardiac disease, regardless of the HRV defining parameter. When defining low HRV by RMSSD <19ms, more patients in this group have hypertension as comorbidity. This group also contains more patients with an ASA classification greater than one.

**Table 1:** Descriptive data of included patients according to pre-specified HRV groups.

	SDNN <20ms (n = 208)	SDNN ≥20ms (n = 220)	p	RMSSD <19ms (n = 233)	RMSSD ≥19ms (n = 195)	p
Heart Rate*	74 [66–84]	68 [60–76]	0.040	77 [67–85]	66 [60–72]	0.001
Age*	68 [62–75]	65 [59–73]	0.002	68 [62–76]	65 [57–72]	<0.001
Age, n(%)			0.039			0.005
< 70 year	111 (53.4)	139 (63.2)		122 (52.4)	128 (65.6)	
≥ 70 year	97 (46.6)	81 (36.8)		111 (47.6)	67 (34.4)	
Sex, n(%)			0.791			0.862
Male	118 (56.7)	121 (55.0)		131 (56.2)	108 (55.4)	
Female	118 (43.3)	99 (45.0)		102 (43.8)	87 (44.6)	
Mean BMI (SD)	25.7 [22.9–28.0]	25.9 [23.0–27.7]	0.041	26.0 [23.3–28.6]	26.0 [23.0–28.1]	0.237
Comorbidities, n(%)						
Cardiac disease	38 (18.2)	15 (6.8)	<0.001	36 (15.6)	17 (8.7)	0.035
Hypertension	78 (37.3)	64 (29.2)	0.075	91 (39.1)	51 (26.2)	0.005
Diabetes Mellitus	27 (12.9)	22 (10.0)	0.351	31 (13.3)	18 (9.2)	0.187
Thyroid disease	9 (4.3)	3 (1.4)	0.066	8 (3.4)	4 (2.1)	0.388
Pulmonary disease	31 (14.9)	21 (9.5)	0.101	30 (12.9)	22 (11.3)	0.604
Vascular disease	16 (7.7)	16 (7.3)	0.901	18 (7.7)	14 (7.2)	0.821
Neurological disease	8 (3.8)	3 (1.4)	0.110	8 (3.4)	3 (1.5)	0.215
Other	20 (9.6)	12 (5.5)	0.111	17 (7.3)	15 (7.7)	0.887
ASA, n(%)			0.208			0.029
ASA-1	55 (26.8)	74 (33.8)		60 (26.1)	69 (35.6)	
ASA-2	138 (67.3)	137 (62.6)		155 (67.4)	120 (61.9)	
ASA-3/4	12 (5.9)	8 (3.7)		15 (6.5)	5 (2.6)	
Localisation tumour, n(%)			0.984			0.357
Right colon	70 (33.5)	74 (33.8)		83 (35.6)	61 (31.3)	
Left colon	79 (37.8)	81 (37.0)		89 (38.2)	71 (36.4)	
Rectum	60 (28.7)	64 (29.2)		61 (26.2)	63 (32.3)	
Tumour stage, n(%)			0.346			0.094
I	48 (23.1)	61 (56.0)		51 (21.9)	58 (29.7)	
II	93 (44.7)	84 (38.2)		106 (45.5)	71 (36.4)	
III	67 (32.2)	75 (34.1)		76 (32.6)	66 (33.8)	
Differentiation grade, n(%)			0.976			0.998
Well/moderate	164 (84.1)	176 (84.2)		186 (84.2)	154 (84.2)	
Poor/undifferentiated	31 (15.9)	33 (15.8)		35 (15.8)	29 (15.8)	

* Non normal-distributed data presented as median with interquartile range.

During a median follow-up of 61 months (IQR 43–89), all cause-death occurred in 101 (23.6%) patients. Cancer recurrence occurred in 83 (19.4%) patients during a median follow-up of 57 months (IQR 39–86).

To rule out any distort in results caused by a delay between ECG and treatment all analyses were repeated after exclusion of ECG’s older than 6 months. This did not lead to any new significant results. Therefore, these results were not displayed in detail in this paper.

### Survival

**Fig 2:**
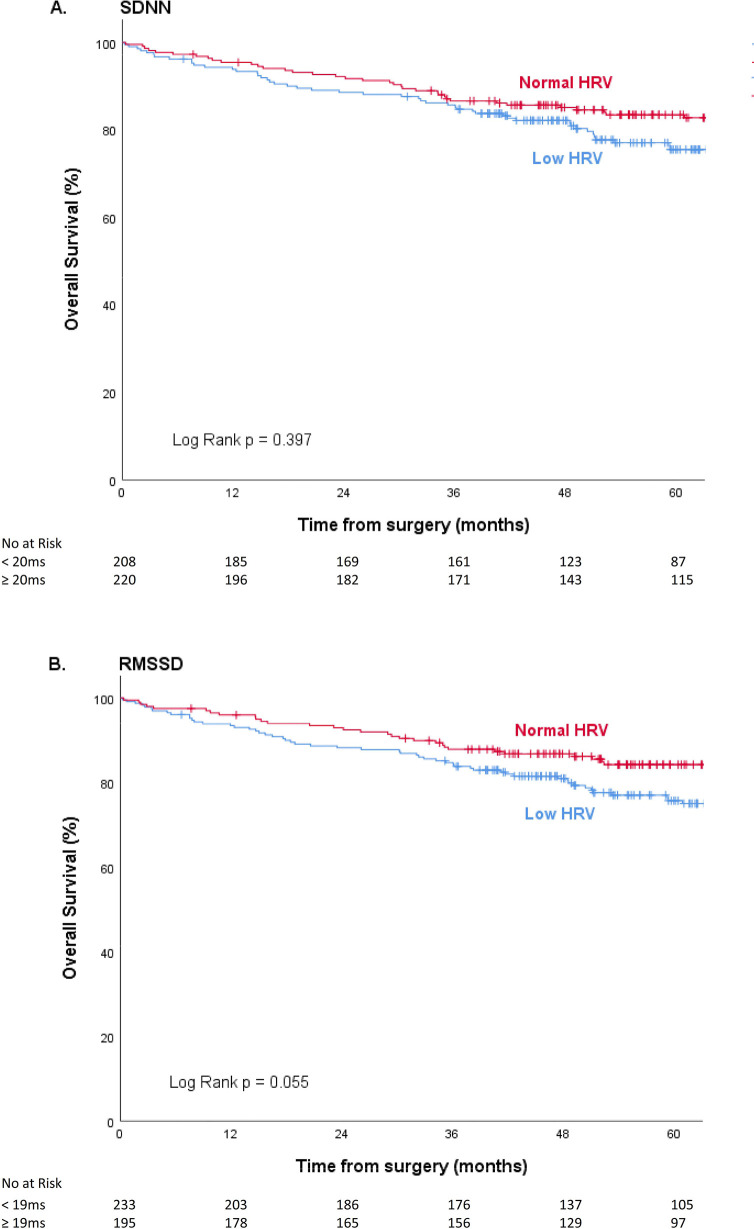
Kaplan-Meier curves for overall survival in groups of low HRV and normal HRV presented as (A) SDNN and (B) RMSSD.

In low HRV groups slightly more patients died compared to normal HRV groups (SDNN <20ms versus ≥20ms, 51 (24.4%) versus 50 (22.8%) respectively; RMSSD <19ms versus ≥19ms, 63 (27.0%) versus 38 (19.5%) respectively). These observed differences between HRV groups in overall survival were not significant (Fig 2; (A) SDNN p = 0.397, (B) RMSSD p = 0.055). After adjustment for some potential confounders these associations remained not significant: SDNN <20ms versus ≥20ms; HR = 0.952 (0.607–1.493), p = 0.829 and RMSSD <19ms versus ≥19ms; HR = 1.321 (0.802–2.178), p = 0.274 (Tables 2 and 3). Age, cardiac disease, tumour stage and post-operative complications had a significant influence on overall survival. Age also differed at baseline and was identified as a confounder. When defining low and normal HRV-groups by SDNN, cardiac disease was identified as confounder. When conducting sensitivity analyses with SDNN and RMSS as continuous variables, results remained the same. There was no association between HRV and overall survival (SDNN; HR = 1.003 (0.999–1.008), p = 0.184 and RMSSD; HR = 0.999 (0.993–1.005), p = 0.759).

**Table 2:** Multivariate analyses of associations of SDNN and covariates with overall and cancer free survival.

	Overall survival		Cancer free survival	
	HR (95% CI)	p	HR (95% CI)	p
SDNN				
<20ms	0.952 (0.607–1.493)	0.829	0.976 (0.599–1.592)	0.924
≥20ms	1 (reference)		1 (reference)	
Heart Rate	1.014 (0.997–1.031)	0.115	1.018 (1.001–1.036)	0.041
Age	1.050 (1.024–2.047)	<0.001	1.000 (0.975–1.026)	0.986
BMI	0.961 (0.909–1.015)	0.151	0.975 (0.917–1.037)	0.426
Cardiac disease				
No	1 (reference)		1 (reference)	
Yes	1.691 (0.953–3.002)	0.073	1.331 (0.661–2.680)	0.423
Hypertension				
No	1 (reference)		1 (reference)	
Yes	0.742 (0.450–1.223)	0.242	0.924 (0.534–1.599)	0.779
ASA-classification				
ASA-1	1 (reference)			
ASA-2	1.289 (0.710–2.340)	0.405	1.441 (0.793–2.617)	0.029
ASA-3/4	0.979 (0.350–2.736)	0.968	1.376 (0.416–4.552)	1.376
Localisation				
Right colon	1 (reference)		1 (reference)	
Left colon	0.689 (0.392–1.211)	0.195	0.741 (0.406–1.350)	0.327
Rectum	1.107 (0.654–1.874)	0.705	1.419 (0.805–2.503)	0.227
Tumour stage				
I	1 (reference)		1 (reference)	
II	1.319 (0.691–2.518)	0.402	2.428 (1.098–5.416)	0.029
III	2.268 (1.215–4.233)	0.010	4.411 (2.038–9.547)	<0.001
Post-operative complication				
No	1 (reference)		1 (reference)	
Yes	2.270 (1.428–3.609)	0.001	1.330 (0.823–2.151)	0.244

**Table 3:** Multivariate analyses of associations of RMSSD and covariates with overall and cancer free survival.

	Overall survival		Cancer free survival	
	HR (95% CI)	p	HR (95% CI)	p
RMSSD				
<19ms	1.321 (0.802–2.178)	0.274	1.192 (0.706–2.011)	0.511
≥19ms	1 (reference)		1 (reference)	
Heart Rate	1.011 (0.993–1.029)	0.229	1.016 (0.997–1.034)	0.093
Age	1.049 (1.022–1.076)	<0.001	0.999 (0.975–1.025)	0.967
BMI	0.959 (0.908–1.013)	0.137	0.972 (0.913–1.035)	0.373
Cardiac disease				
No	1 (reference)		1 (reference)	
Yes	1.615 (0.907–2.875)	0.104	1.283 (0.643–2.557)	0.480
Hypertension				
No	1 (reference)		1 (reference)	
Yes	0.742 (0.450–1.223)	0.241	0.932 (0.539–1.612)	0.802
ASA-classification				
ASA-1	1 (reference)		1 (reference)	
ASA-2	1.309 (0.721–2.375)	0.337	1.466 (0.809–2.658)	0.207
ASA-3/4	0.944 (0.339–2.631)	0.913	1.350 (0.410–4.450)	0.622
Localisation				
Right colon	1 (reference)		1 (reference)	
Left colon	0.685 (0.391–1.201)	0.187	0.735 (0.404–1.338)	0.314
Rectum	1.122 (0.664–1.896)	0.667	1.426 (0.810–2.510)	0.219
Tumour stage				
I	1 (reference)		1 (reference)	
II	1.136 (0.609–2.117)	0.689	2.428 (1.095–5.386)	0.029
III	2.090 (1.152–3.793)	0.015	4.402 (2.034–9,526)	<0.001
Post-operative complication				
No	1 (reference)		1 (reference)	
Yes	2.237 (1.405–3.560)	0.001	1.313 (0.812–2.125)	0.267

**Fig 3:**
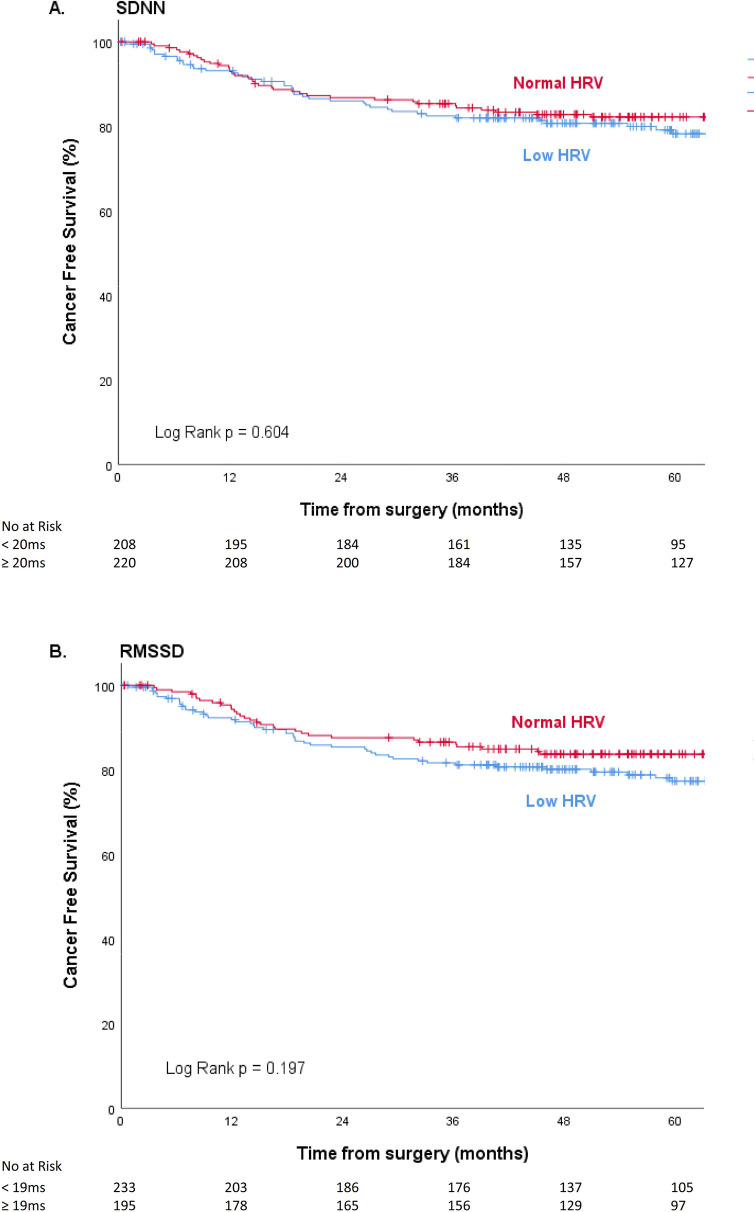
Kaplan-Meier curves for cancer free survival in groups of low HRV and normal HRV presented as (A) SDNN and (B) RMSSD.

In low HRV groups slightly more patients had recurrence of cancer compared to normal HRV groups (SDNN <20ms versus ≥20ms, 42 (20.1%) versus 41 (18.7%) respectively; RMSSD <19ms versus ≥19ms, 50 (21.5%) versus 33 (16.9%) respectively). These observed differences between HRV groups in cancer free survival were not significant (Fig 3; (A) SDNN p = 0.604, (B) RMSSD p = 0.197. As in overall survival, after adjustment for some potential confounders these associations remained not significant: SDNN <20ms versus ≥20ms; HR = 0.976 (0.599–1.592), p = 0.924 and RMSSD <19ms versus ≥19ms; HR = 1.192 (0.706–2.011), p = 0.511 (Tables 2 and 3). In SDNN-based groups baseline heart rate was identified as a confounding variable. Sensitivity analyses with SDNN and RMSSD as continuous variables did not alter these results. There was no association between HRV and cancer free survival (SDNN; HR = 1.001 (0.995–1.007), p = 0.791 and RMSSD; HR = 0.991 (0.979–1.002), p = 0.120).

### CEA-level

CEA-level at baseline and one year check-up was registered in 371 patients and elevated in 88 of these patients. This was elevated at one year check-up in only 37 patients. Low HRV was not significantly associated with elevated CEA-levels at one year check-up, as shown in Table 4. Sensitivity analyses with SDNN and RMSSD as continuous variables did not alter these results (SDNN; HR = 0.999 (0.989–1.009), p = 0.821 and RMSSD; HR = 0.995 (0.981–1.008), p = 0.437). Adjusting for covariates was not possible because of the small number of patients with an elevated CEA-level.

**Table 4:** Univariate analyses of low HRV and risk of elevated CEA-level at one year check-up.

	CEA > 5.0 μg/l			CEA > 5.0 μg/l	
	OR (95% CI)	p		OR (95% CI)	p
**Independent of baseline CEA**		0.397	**Independent of baseline CEA**		0.590
SDNN <20ms (n = 20, 11.4%)	1.342 (0.679–2.653)		RMSSD <19ms (n = 21, 10.8%)	1.207 (0.608–2.394)	
SDNN ≥20ms (n = 17, 8.7%)	1 (reference)		RMSSD ≥19ms (n = 16, 9.1%)	1 (reference)	
**Normal baseline CEA ≤ 5.0 μg/l**		0.212	**Normal baseline CEA ≤ 5.0 μg/l**		0.357
SDNN <20ms (n = 9, 6.5%)	2.451 (0.600–10.015)		RMSSD <19ms (n = 8, 5.2%)	0.521 (0.130–2.085)	
SDNN ≥20ms (n = 6, 4.2%)	1 (reference)		RMSSD ≥19ms (n = 7, 5.5%)	1 (reference)	
**Elevated baseline CEA > 5.0 μg/l**		0.964	**Elevated baseline CEA > 5.0 μg/l**		0.232
SDNN <20ms (n = 11, 29.7%)	1.027 (0.316–3.337)		RMSSD <19ms (n = 13, 32.5%)	2.055 (0.631–6.695)	
SDNN ≥20ms (n = 11, 21.6%)	1 (reference)		RMSSD ≥19ms (n = 9, 18.7%)	1 (reference)	

**Fig 4:**
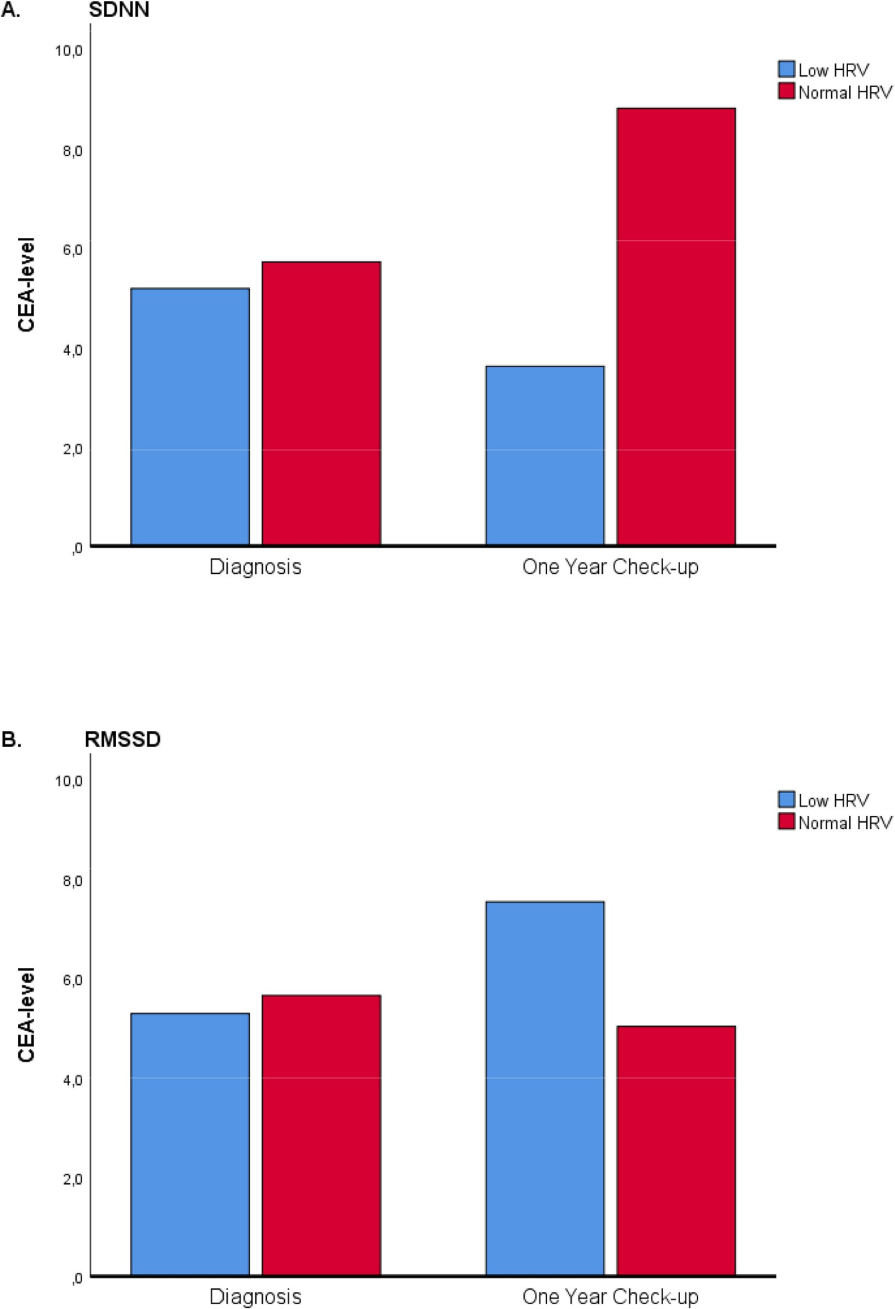
Bar chart for CEA-level at baseline and one year check-up in groups of low HRV and normal HRV presented as (A) SDNN and (B) RMSSD.

Differences between CEA-level at baseline and one year check-up were compared between and within HRV-groups. Results were displayed in Fig 4. There were no significant differences in CEA-level at baseline and one year check-up between the low HRV group and normal HRV group defined by SDNN and RMSSD, p = 0.297 and p = 0.521 respectively. Also, there were no significant differences in CEA-level at baseline and at one year check-up within the low HRV group and normal HRV group defined by SDNN and RMSSD, p = 0.733 and p = 0.718 respectively.

### Post-operative complications

In 187 patients one or more postoperative complications occurred within 30 days after surgery. The occurrence of a post-operative complication was not significantly associated with low HRV defined as SDNN <20ms or RMSSD <19ms, even after adjustment for some potential confounders (Table 5). Heart rate, age, cardiac disease and hypertension were identified as confounding covariates. When conducting sensitivity analyses with SDNN and RMSS as continuous variables the association between occurrence of a post-operative complication with baseline HRV remained not significant (SDNN; HR = 1.004 (0.999–1.010), p = 0.085 and RMSSD; 1.002 (0.997–1.007), p = 0.377). The same applied when postoperative complications were graded according to the Clavien-Dindo classification and the complication that is graded the highest for each patient is compared to the absence of postoperative complications (Table 6).

**Table 5:** Low HRV and risk of occurrence of a post-operative complication within 30 days.

	Post-operative complication	
	OR (95% CI)	p
Unadjusted		0.313
SDNN <20ms (n = 96, 46.2%)	1.218 (0.830–1.785)	
SDNN ≥20ms (n = 91, 41.4%)	1 (reference)	
Adjusted*		0.859
SDNN <20ms (n = 96, 46.2%)	1.038 (0.689–1.563)	
SDNN ≥20ms (n = 91, 41.4%)	1 (reference)	
Unadjusted		0.261
RMSSD <19ms (n = 108, 46.4%)	1.247 (0.849–1.832)	
RMSSD ≥19ms (n = 79, 40.5%)	1 (reference)	
Adjusted*		0.844
RMSSD <19ms (n = 108, 46.4%)	1.044 (0.677–1.611)	
RMSSD ≥19ms (n = 79, 40.5%)	1 (reference)	

*adjusted for heart rate, age, cardiac disease and hypertension.

**Table 6:** Low HRV and severity of post-operative complication(s) according to Clavien-Dindo classification.

		Minor (grade I and II)			Major (grade III, IV, V)	
		OR (95% CI)	p		OR (95% CI)	p
Unadjusted			0.333			0.552
SDNN <20ms	(n = 60, 28.8%)	1.245 (0.798–1.943)		(n = 36, 17.3%)	1.174 (0.692–1.994)	
SDNN ≥20ms	(n = 55, 25.0%)	1 (reference)		(n = 36, 16.4%)	1 (reference)	
Adjusted*			0.581			0.712
SDNN <20ms	(n = 60, 28.8%)	1.141 (0.715–1.819)		(n = 36, 17.3%)	1.109 (0.641–1.917)	
SDNN ≥20ms	(n = 55, 25.0%)	1 (reference)		(n = 36, 16.4%)	1 (reference)	
Unadjusted			0.526			0.208
RMSSD <19ms	(n = 64, 27.5%)	1.155 (0.740–1.805)		(n = 44, 18.9%)	1.414 (0.825–2.423)	
RMSSD ≥19ms	(n = 51, 26.2%)	1 (reference)		(n = 28, 14.4%)	1 (reference)	
Adjusted*			0.926			0.345
RMSSD <19ms	(n = 64, 27.5%)	0.977 (0.594–1.605)		(n = 44, 18.9%)	1.330 (0.736–2.406)	
RMSSD ≥19ms	(n = 51, 26.2%)	1 (reference)		(n = 28, 14.4%)	1 (reference)	

*adjusted for heart rate, age categories (<70 vs ≥70) and hypertension.

## Discussion

In this observational cohort-study we determined the Heart Rate Variability in pre-operative ECGs of 428 patients with primary colorectal cancer who underwent curative surgical treatment. HRV refers to physiological variations in beat-to-beat intervals. It was presented in time-domain parameters; SDNN and RMSSD. HRV was not significantly associated with overall survival or cancer free survival, independent of some risk factors. Also, this study showed no significant differences in CEA-levels at one year check-up between patients with low HRV and patients with normal HRV. Patients with low HRV did not have more, or more severe, post-operative complications compared to patients with normal HRV.

Tumorigenesis has three fundamental mechanisms: (1) inflammation promoting oxidative stress and stimulating tumour growth, (2) oxidative stress causing DNA-damage and interfering with subsequent repair mechanisms and (3) sympathetic neurotransmitters stimulating tumour metastasis and progression [5,7]. It has been suggested that afferent fibres of the vagus nerve inform the brain about peripheral inflammation. This is followed by a brain-to-immune response via the efferent route of the vagus nerve that modulates the function of immune-regulatory cells through the release of neurotransmitters via the cholinergic anti-inflammatory pathway. Malfunctioning of this route may play part in the onset of cancer [10]. This theory has been supported by studies of patients with gastric ulcers who underwent a vagotomy, who then showed an increased risk of developing lung- and colorectal cancer [25–27]. The vagus nerve is also believed to modulate tumour progression. An active vagus nerve can inhibit inflammation, oxidative stress and the release of sympathetic neurotransmitters that stimulate tumour metastasis and progression [10–11]. Absence of this inhibitory effect in turn results in metastasis and tumour progression, as shown in a study of *Erin et al*. showing that mice who underwent chemical vagotomy developed more metastasis of breast-cancer cells than controls [28]. Epidemiologically, low HRV has been associated with worse overall survival over time in patients with recurrent or metastatic cancer of various types, even after correction for confounders [13,24,29–35]. However, the results of the present study do not support these findings.

To our knowledge, this is the first study including only patients with colorectal cancer who are eligible for curative treatment by partial bowel resection, and not those receiving palliative treatment. *De Couck et al*. studied the relationship between HRV and tumour burden in both curative and palliative patients with prostate cancer or non-small cell lung cancer. Independent of confounders, the hypothesised inverse relationship of HRV and the tumour marker PSA at 6 and 24 months after diagnosis was only significant in patients with stage IV prostate cancer, not stage II and III [24]. In colorectal cancer *Mouton et al*. found that low HRV defined as SDNN < 20 ms predicted significantly higher levels of the tumour marker CEA at 12 months after diagnosis. Again, these results were only found in patients receiving palliative treatment, not curative [14]. Only one previous study showed a significant inverse relationship between HRV and mortality in cancer in general, independent of stage [4]. This study of *Guo et al*. had a large study population of 651 patients with various types of cancer. Low HRV was defined as SDNN < 70ms and was significantly associated with shorter survival times. This suggests that HRV is a predictive indicator of survival time not only in palliative- but also in curative patients. However, results were not controlled for cancer type which could affect both HRV and survival, and should therefore be interpreted with caution [10]. The fact that the patients recruited in the present sample were only with less advanced cancer, may partly explain the lack of prognostic role in HRV in this sample.

Some of these previous studies suggest a bidirectional relationship between vagus nerve activity and cancer [7]. However, based on current evidence on this subject we cannot support this hypothesis. The positive association between low HRV and worse prognosis found in patients with colorectal cancer receiving palliative treatment, but not in patients receiving curative treatment suggest that this relation is not bidirectional, but that advanced cancer is associated with low HRV. Mid-late-stage tumours are often accompanied by damage to autonomic nerves resulting in decreased HRV [5]. A study of De Couck et al. showed that cancer patients in general have a significantly lower HRV than healthy people [7]. The same results were found in studies of Bijoor et al. where RMSSD was found to be significantly lower in patients with early- and advanced stage cancer compared to a healthy control group [36,37]. When comparing patients with advanced stage cancer (TNM III and IV) to patients with an early stage of cancer (TNM I and II), RMSSD was found to be significantly lower in patients with advanced stages of cancer [36,37]. Thus, though experimental studies in animals show that vagal nerve functioning can causally slow tumorigenesis, the human data suggests that the malignant tumour causes vagal nerve dysfunction and therewith decreased HRV [38].

Besides the proposed influence of low HRV on survival of colorectal cancer patients through development and increased progression of cancer, Reimer et al. suggested that low HRV could influence survival of those undergoing surgical treatment due to more- and more severe post-operative complications [15]. However, the results found in this study were not concurrent with those of Reimer et al. who included 53 patients with ASA > 1 undergoing elective surgery. Their analysis of HRV was through power-spectrum parameters based on longer ECG-recordings instead of the time-domain parameters based on 10s ECGs used in this study. But the difference in used parameters used in both studies is probably not the explanation for the differences in results between both studies, since it has been demonstrated that RMSSD and SDNN based on ECG recordings of 10s are in substantial agreement with those of 45min and a 10s ECG therefore suffices to determine time domain HRV parameters. However, Reimer et al. did not correct for possible confounders. In their study patients with low HRV were more likely to have diabetes, a known risk factor for postoperative ileus and wound infections, both found to be common postoperative complications in their low HRV group. Correcting for this comorbidity may change the significance of their findings [39,40]. A study of Scheffler et al. did not show any significant pre-operative differences in HRV between patients with or without post-operative complications, or between patients with post-operative complications of different severity levels [41]. Thus, also in relation to predicting post-operative complications, results are not uniform.

This study was subject to a number of limitations. Due to its retrospective character confounding may have occurred. We attempted to correct for all possible confounders within database availability with regression analyses. Also, not all possible confounding factors were within database availability. Reasons for diagnostic procedure, smoking, alcohol abuse are presumed to have an effect on HRV and outcomes, but were not documented in enough patients to be taken into account when conducting multivariate analyses. Analysed ECGs were those performed before or at time of pre-operative screening. In this cohort 85% ECG’s were made within the year before surgery. Only 9 ECG’s were older than five years at time of surgery. The precise effects of these differences in time from ECG to cancer treatment on HRV are unknown. To test the implication of a longer time between ECG and treatment we repeated all analyses after excluding patients with an ECG older than 6 months. This did not alter the results, which could be explained by the theory that low HRV causes cancer and the amount of time it takes for colorectal tumours to develop [42]. A large amount of patients was excluded from this study due to cardiac arrhythmias. This could potentially have led to attenuation of the association between HRV and survival in this study population compared to the actual population, because theoretically these cardiac arrhythmias could be originated from an inflammatory state due to cancer which would have let to lowering of HRV in this group and these cardiac arrhythmias are presumed to be associated with higher mortality [43]. In our dataset five year follow-up was not yet complete for all patients at time of analysis, resulting in declining numbers at risk during the years of follow-up. To determine cancer free survival, patients dying without cancer recurrence were censored at time of death which results in a lower statistical power over the years of follow-up.

There are also some important strengths to the study like the entirety of variables in this dataset. The number of included patients was adequate to conduct analyses adjusted for multiple covariates. The clinical implementation of HRV analysis is easy and non-invasive. The ECG conducted for pre-operative screening can be used for analyses of HRV.

## Conclusion

In the first study to assess the prognostic value of HRV in patients with primary colorectal cancer who underwent curative surgical treatment, HRV was not associated with overall or cancer free survival. Furthermore, low HRV was not significantly associated with elevated CEA-levels during follow-up or with more- or more severe post-operative complications. Hence, this study does not suggest any expected benefit from HRV-improving interventions to improve clinical outcomes in patients with early colorectal cancer.
